# Are pension types associated with happiness in Japanese older people?: JAGES cross-sectional study

**DOI:** 10.1371/journal.pone.0197423

**Published:** 2018-05-21

**Authors:** Ichiro Sasaki, Katsunori Kondo, Naoki Kondo, Jun Aida, Hiroshi Ichikawa, Takashi Kusumi, Naoya Sueishi, Yuichi Imanaka

**Affiliations:** 1 Department of Healthcare Economics and Quality Management, Graduate School of Medicine, Kyoto University, Kyoto, Japan; 2 Department of Social Preventive Medical Sciences, Center for Preventive Medical Sciences, Chiba University, Chiba, Japan; 3 Department of Gerontological Evaluation, Center for Gerontology and Social Science, National Center for Geriatrics and Gerontology, Aichi, Japan; 4 Department of Health and Social Behavior, School of Public Health, The University of Tokyo, Tokyo, Japan; 5 Department of International and Community Oral Health, Tohoku University Graduate School of Dentistry, Sendai, Japan; 6 Department of Medical Life Systems, Graduate School of Life and Medical Sciences, Doshisha University, Kyoto, Japan; 7 Division of Cognitive Psychology in Education, Graduate School of Education, Kyoto University, Kyoto, Japan; 8 Graduate School of Economics, Kobe University, Kobe, Japan; Cardiff University, UNITED KINGDOM

## Abstract

**Background:**

Although many previous studies have examined the determinants of happiness in older adults, few have investigated the association between pension types and happiness. When compared to other conventional socioeconomic indicators, pension types may be more indicative of long-term socioeconomic status as they can reflect a person’s job history over their life course. This study examined the association between pension types and happiness in Japanese older people.

**Methods:**

Cross-sectional survey data from the Japan Gerontological Evaluation Study were used to analyze the association between pension types and happiness. The study population comprised 120152 participants from 2013. We calculated the prevalence ratios of happiness for the different pension types using Poisson regression models that controlled for age, sex, marital status, equivalent income, wealth, education level, working status, occupation, depression, and social support.

**Results:**

After controlling for socioeconomic indicators, the prevalence ratios (95% confidence intervals) of happiness for no pension benefits, low pension benefits, and moderate pension benefits relative to high pension benefits were 0.77 (0.73–0.81), 0.95 (0.94–0.97), and 0.98 (0.97–0.99), respectively. However, the inclusion of depression as a covariate weakened the association between pension types and happiness.

**Conclusions:**

While pension types were associated with happiness after adjusting for other proxy measures of socioeconomic status, the association diminished following adjustment for depression. Pension types may provide rich information on socioeconomic status and depression throughout the course of life. In addition to conventional socioeconomic indicators, pension types should also be considered when assessing the determinants of happiness in older adults.

## Introduction

Identifying the determinants of happiness is not only important for improving human welfare, but also provides insight into factors that affect health and longevity. The recognition of these determinants and the implementation of appropriate measures may lead to elevated health statuses, increased life expectancy, and lower medical expenses.

Previous studies on the determinants of happiness have mainly focused on demographic and socioeconomic status (SES) factors, health status, longevity, health insurance, and social support. Among these factors, happiness has been reported to be associated with high income and high education levels [1–4], self-perceived health and longevity [5–9], health insurance coverage and access to medical care [10,11], and robust social support systems [12,13]. However, few studies have examined the relationship between happiness and pensions [14,15]. Furthermore, those studies have generally focused on participation in pension systems or coverage, and little is known about the association between pension types and happiness. In this study, as determinants of happiness in older adults, we emphasized income (about 70% of which is pension for Japanese older adults) and health (especially mental health, such as depression, as it affects all aspects of life) [16]. Depressive symptoms were shown to be associated with low happiness [17,18]. In particular, income has become increasingly important for retirees and the elderly. Data from a panel of retirees aged 51–61 in the United States (Health and Retirement Study: HRS), Bender (2004) showed that voluntary retirement, total revenues (including pensions), and health affect satisfaction following retirement [19], and Panis (2003) found that better health and higher income contribute to greater happiness, with pension annuities increasing this sense of happiness and reducing depression [20]. We posited that pension types would be associated with happiness because these may be indicative of SES and depressive symptoms across a person’s course of life in Japan.

As lifespans continue to increase, older adults who rely solely on personal savings for living expenses face an increased risk of exhausting their funds during their lifetimes. Because Japanese public pensions offer pension benefits for the entirety of a person’s life, individuals with higher pension benefits may have a stronger sense of financial security in their old age. In this way, pension types may influence old-age poverty, economic anxiety, and depression. In Japanese older adults, pension benefits account for an average of 70% of total income [21], and 89% of Japanese 65- to 69-year-olds received pension benefits in 2012 [22]. In addition, older persons with high pensions generally receive more than three times the pension benefits as older people with low pensions. It is therefore possible that pension types are indicative of income disparities in older age. By focusing on pension types, we may be able to ascertain not only income level and working status in each subject’s active working period, but also their post-retirement income level. The purpose of this study was to examine the association between pension types and happiness in Japanese older people.

## Methods

### Study design and population

This study used data obtained from the Japan Gerontological Evaluation Study (JAGES) project, which is an on-going prospective multi-wave cohort study that began in 2003. Data from 2013 were utilized for this analysis. The participants of the JAGES project comprise Japanese older adults aged 65 years or older who have not been certified as needing long-term care services. Using a questionnaire-based survey, the JAGES project asks participants about factors associated with health status and its determinants in older age; these factors include health status, depression, happiness, SES, and social capital [23,24]. Explanations of the study and the self-reported questionnaire were sent by mail to the residents. They were informed that participation was voluntary and that returning the self-administered questionnaire would be interpreted as implying consent.

The JAGES survey in 2013 sent questionnaires to 193,694 Japanese older adults from 30 municipalities. Participating municipalities and individuals were those who had previously agreed to participate in the JAGES Survey. Responses were collected from 137,736 respondents (response rate: 71.1%). From these respondents, 7996individuals were excluded because of missing data in age and sex. Another 9588 individuals were excluded because they had failed to provide information on pension types or happiness. Thus, with a final sample size for analysis of 120,152 subjects, our overall coverage rate was 62.0%.

### Dependent variable

Previous studies have generally measured subjective well-being using a single question such as, “Taking all things together, would you say that you are very happy, pretty happy, not too happy, or not happy at all?”. It has been shown that single-item measures of subjective well-being have moderate reliability [25–27]. In this study, we measured self-perceived happiness through the following question: “How would you rate your overall happiness level on a ten-point scale of one (very unhappy) to ten (very happy)?”. This scale was employed because it is the JAGES study's sole measure of self-perceived happiness.

Based on this happiness scale, we constructed a binary variable of happiness to use as the dependent variable in Poisson regression analyses. Happiness was defined as a self-rated score of 7 to 10 points, and unhappiness was defined as a score of 1 to 6 points. This cut-off point was determined using a previously conducted survey that found that the average happiness score in Japanese older people was 6 points [28].

### Pension types

Japanese pension systems can be categorized into three main types according to differences in occupation and working status between the ages of 20 to 60 years. These three pension types are the “National Pension Plan”, “Employees’ Pension and Mutual-Aid Society Pension Plan”, and “Corporate Pension Plan”; for simplicity, these are referred to as “NA Pension”, “EM Pension”, and “CP Pension”, respectively. People who did not participate in any pension plan or did not pay pension fees between the ages of 20 to 60 years do not receive any pension benefits in old age.

The pension types are determined by working-age occupation and employment type across a person’s life in Japan, and are therefore indicative of working-age income levels. NA Pension is the basic pension plan for all pension beneficiaries, and constitutes the first pension pillar in Japan. Beneficiaries who receive only NA Pension benefits are the self-employed, non-regular workers, and the unemployed. EM Pension represents the second pension pillar. The beneficiaries of EM Pension had generally worked as regular employees in small and medium-sized businesses or as civil servants, and receive both NA Pension and EM Pension benefits. The beneficiaries of CP Pension, which is the third pension pillar, receive CP Pension benefits in addition to NA Pension and EM Pension benefits. These beneficiaries had generally worked as regular employees in large corporations, and their pension benefits are the highest among the three pension types. The mean amounts of pension benefits received are, in decreasing order, CP Pension (>0.15 million Japanese yen), EM Pension (approximately 0.15 million Japanese yen), and NA Pension (approximately 0.05 million Japanese yen). Because pension benefits account for approximately 70% of all income for Japanese older people, pension types are a major contributing factor to an individual’s economic status.

### Geriatric Depression Scale

Similar to Sun et al (2016) and Graham et al (2011), we considered depression as one dimension for assessing quality of life related to health. To assess depressive symptoms in older adults, we used the Japanese version of the 15-item Geriatric Depression Scale (GDS) [29]. The GDS score ranges from 0 to 15, with higher scores indicating more severe symptoms. The correlation between happiness in our 10-point scale and the 15-point GDS scale was -0.584. Although this correlation was fairly high, as it failed to achieve -0.7 and as happiness did not equal GDS, we included GDS as a control variable.

We used a score of 5 and 10 as cut-off values for moderate and severe depression, respectively, in accordance with the results of a previous validation study conducted in Japan.[29]

### Covariates

Demographic variables included sex, age, and marital status. Age was categorized into the following four categories: 65–69 years, 70–79 years, 80–89 years, and ≥90 years. Marital status was categorized into married, widowed, divorced, never married, and others. SES indicators included equivalent income (<2.00 million yen, 2.00–3.99 million yen, and ≥4.00 million yen) [30–32], wealth (<0.50 million yen, 0.50–0.99 million yen, 1.00–4.99 million yen, 5.00–9.99 million yen, 10.00–49.99 million yen, and ≥50.00 million yen), education level (≤9 years, 10–12 years, and ≥13 years of formal education), and working status (currently working, retired, or never worked). Equivalent income was defined as the income divided by square of number of household members. Occupation was grouped into specialist/technician/manager, clerical worker/sales/service jobs, labor, agriculture/forestry or fisheries, self-employed, other and unemployed. Social support was analyzed as the receipt or provision of emotional support and instrumental support.

### Statistical analysis

Because the prevalence of unhappiness was 32.4% (greater than 10%), odds ratios with logistic regression would tend to be overestimated [33]. We therefore performed Poisson regression analyses with robust variance estimators to calculate the adjusted prevalence ratios (PRs) and 95% confidence intervals (CIs) of happiness for the different pension types after controlling for sex, age, marital status, SES, GDS, and social support [34,35] Subjects who did not provide information on the independent variables (except for pension type) were assigned to missing data categories. The outcome variable of happiness was analyzed as a binary variable (happy: score of 7 to 10; unhappy: 1 to 6).

We constructed three models with an increasing number of covariates. In Model 1, we examined the associations between pension types and happiness after controlling for sex, age, marital status, and social support. In Model 2, we also included the SES indicators (equivalent income, wealth, education level, working status, and occupation) to clarify whether the associations between pension types and happiness remained consistent even after controlling for these factors. In Model 3, we further included depressive symptoms to examine if depression functioned as a mediating variable. All statistical analyses were performed using SPSS version 24.0 software.

### Ethics approval

Approval for this study was obtained from the Ethics Committee of Nihon Fukushi University and the Ethics Committee of Kyoto University Graduate School of Medicine.

## Results

Table 1 shows the subjects’ characteristics. Of the 120152 subjects, 46.9% were men. The mean (standard deviation) age was 73.9 years (6.2). Approximately 67.6% of the sample perceived themselves as being happy (happiness score ≥7). Regarding the pension types, the proportions of individuals with no pension (zero benefits), NA Pension benefits (low), EM Pension benefits (moderate) and CP Pension benefits (high) were 1.4%, 29.4%, 58.3% and 10.9%, respectively. Of the subjects, 62.0%, 16.3% and 5.5% reported having no depression (GDS <5), moderate depression (5 ≤ GDS < 10) and severe depression (GDS ≥10), respectively. The mean happiness score was 7.3 (standard deviation: 1.9).

**Table 1:** Descriptive characteristics of the study subjects(N = 120152).

	Total N	%	Happy N	%	Unhappy N	%	p-value
Sex							
Male	56334	46.9	36812	45.3	19522	50.2	<0.001
Female	63818	53.1	44421	54.7	19397	49.8	
Age							
65–69	34041	28.3	23280	28.7	10761	27.6	<0.001
70–79	62909	52.4	41688	51.3	21221	54.5	
80–89	21727	18.1	15211	18.7	6516	16.7	
≥90	1475	1.2	1054	1.3	421	1.1	
Marital Status							
Married	85505	71.2	59955	73.8	25550	65.6	<0.001
Widowed	24503	20.4	16165	19.9	8338	21.4	
Divorced	4044	3.4	1952	2.4	2092	5.4	
Never married	2672	2.2	1285	1.6	1387	3.6	
Others	1033	0.9	464	0.6	569	1.5	
Missing	2395	2.0	1412	1.7	983	2.5	
Equivalent income							
<2.00 million	51654	43.0	30625	37.7	21029	54.0	<0.001
2.00–3.99 million yen	38177	31.8	28624	35.2	9553	24.5	
4.00 million yen or above	10727	8.9	9164	11.3	1563	4.0	
Missing	19594	16.3	12820	15.8	6774	17.4	
Wealth							
<0.5 million	5210	4.3	2299	2.8	2911	7.5	<0.001
0.50–0.99 million yen	4803	4.0	2445	3.0	2358	6.1	
1.00–4.99 million yen	13761	11.5	8106	10.0	5655	14.5	
5.00–9.99 million yen	16179	13.5	10206	12.6	5973	15.3	
10.00–49.99 million yen	38652	32.2	28845	35.5	9807	25.2	
≧50.00 million yen	14328	11.9	12199	15.0	2129	5.5	
Missing	27219	22.7	17133	21.1	10086	25.9	
Education							
≦9 years	49094	40.9	30382	37.4	18712	48.1	<0.001
10–12 years	44625	37.1	31042	38.2	13583	34.9	
≧13 years	24576	20.5	18641	22.9	5935	15.2	
Missing	1857	1.5	1168	1.4	689	1.8	
Working Status							
Never worked	13856	11.5	9358	11.5	4498	11.6	<0.001
Working now	27238	22.7	19058	23.5	8180	21.0	
Retired	71197	59.3	47890	59.0	23307	59.9	
Missing	7861	6.5	4927	6.1	2934	7.5	
Occupation							
Profession,Engineer,Manager	26039	21.7	18916	23.3	7123	18.3	<0.001
Clerical worker/sales/service bobs	37241	31.0	25943	31.9	11298	29.0	
Labor	15932	13.3	9694	11.9	6238	16.0	
Agriculture,forestry or fisheries/self-employed	12059	10.0	8020	9.9	4039	10.4	
Others	10939	9.1	6709	8.3	4230	10.9	
Never worked	6014	5.0	4253	5.2	1761	4.5	
Missing	11928	9.9	7698	9.5	4230	10.9	
Pension Types							
CP Pension(Rich)	13047	10.9	9480	11.7	3567	9.2	<0.001
EM Pension(Moderate)	70071	58.3	47543	58.5	22528	57.9	
NA Pension(Poor)	35307	29.4	23468	28.9	11839	30.4	
No Pension(Zero)	1727	1.4	742	0.9	985	2.5	
GDS							
No depression(GDS<5)	74480	62.0	60290	74.2	14190	36.5	<0.001
Moderate depression(5≦GDS<10)	19555	16.3	8217	10.1	11338	29.1	
Depression(≧10)	6637	5.5	823	1.0	5814	14.9	
Missing	19480	16.2	11903	14.7	7577	19.5	
Receiving emotional support							
Present	111119	92.5	76944	94.7	34175	87.8	<0.001
Absent	6398	5.3	2657	3.3	3741	9.6	
Missing	2635	2.2	1632	2.0	1003	2.6	
Providing emotional support							
Present	107575	89.5	74824	92.1	32751	84.2	<0.001
Absent	8459	7.0	3909	4.8	4550	11.7	
Missing	4118	3.4	2500	3.1	1618	4.2	
Receiving instrumental support							
Present	111930	93.2	77745	95.7	34185	87.8	<0.001
Absent	5608	4.7	1976	2.4	3632	9.3	
Missing	2614	2.2	1512	1.9	1102	2.8	
Providing instrumental support							
Present	91844	76.4	63979	78.8	27865	71.6	<0.001
Absent	21765	18.1	13083	16.1	8682	22.3	
Missing	6543	5.4	4171	5.1	2372	6.1	
Happiness Score							
1 point	1104	0.9	0	0.0	1104	2.8	<0.001
2 points	833	0.7	0	0.0	833	2.1	
3 points	2071	1.7	0	0.0	2071	5.3	
4 point	2716	2.3	0	0.0	2716	7.0	
5 points	17149	14.3	0	0.0	17149	44.1	
6 points	15046	12.5	0	0.0	15046	38.7	
7 points	20228	16.8	20228	24.9	0	0.0	
8 points	31401	26.1	31401	38.7	0	0.0	
9 points	12664	10.5	12664	15.6	0	0.0	
10 points	16940	14.1	16940	20.9	0	0.0	
Happiness							
Happy (Happiness score≧7)	81233	67.6	81233	100.0	0	0.0	<0.001
Unhappy (Happiness score<7)	38919	32.4	0	0.0	38919	100.0	

Abbreviation: GDS, Geriatric Depression Scale.

The JAGES dataset is not a representative sample of elderly people in Japan, so to validate it for use in this study, we compared the values for each of the variables, age, sex, and wealth, with corresponding values from representative samples of elderly people throughout Japan. It was indicated that the values for these variables from our sample corresponded well with those of representative samples [36,37].

Table 2 shows the differences in SES, depressive symptoms, and happiness according to pension type. Among the subjects with high CP Pension benefits, there were higher proportions of individuals who were happy. These individuals also tended to have a higher equivalent income (≥4.00 million yen), higher education level (≥13 years of formal education), and no depression.

**Table 2:** Study variables according to pension type (n = 120152).

		Pension types					
		No Pension	NA Pension	EM Pension	CP Pension	p-value	Pearson's chi-square value
		(Zero)	(Poor)	(Mode)	(Rich)		
SES	Equivalent income						
	<2.00 million	55.5	46.5	43.4	29.7	<0.001	5186.168
	2.00–3.99 million yen	11.9	23.3	33.4	48.8		
	4.00 million yen or above	5.3	7.5	8.8	13.9		
	Missing	27.3	22.7	14.4	7.6		
	Education						
	≦9 years	50.9	49.9	39.1	24.5	<0.001	3456.290
	10–12 years	30.3	34.3	37.6	43.4		
	≧13 years	16.2	14.0	21.8	31.0		
	Missing	2.5	1.8	1.4	1.1		
	Working Status						
	Never worked	19.1	23.7	6.7	3.5	<0.001	13880.457
	Working now	29.3	25.2	21.0	24.3		
	Retired	41.6	38.5	67.9	71.0		
	Missing	10.0	12.6	4.4	1.3		
	Occupation						
	Profession,Engineer,Manager	14.2	9.3	24.9	38.8	<0.001	20172.543
	Clerical worker/sales/service jobs	29.8	26.7	33.1	31.4		
	Labor	10.9	6.1	16.3	16.7		
	Agriculture, forestry or fisheries /self-employed	9.6	21.9	5.6	2.0		
	Others	15.3	9.9	9.3	5.3		
	Never worked	8.6	11.6	2.4	0.7		
	Missing	11.5	14.5	8.5	5.0		
	the number of employees at the company or organization where they worked the longest						
	1–499	65.6	48.8	62.1	41.8	<0.001	27613.375
	500–9999	2.8	4.6	16.7	32.4		
	10000 over	1.0	1.8	8.5	21.2		
	unknown	9.1	6.4	5.1	2.2		
	Never worked	9.3	15.3	2.4	0.8		
	Missing	12.2	23.1	5.2	1.6		
GDS	No depression(GDS<5)	41.3	56.8	63.2	72.3	<0.001	1796.081
	Moderate depression(5≦GDS<10)	23.9	16.8	16.3	13.9		
	Depression (≧10)	16.1	6.1	5.3	3.8		
	Missing	18.6	20.3	15.3	10.0		
Happiness	Happy (Happiness score≧7)	43.0	66.5	67.8	72.7	<0.001	653.803
	Unhappy (Happiness score<7)	57.0	33.5	32.2	27.3		

Abbreviations: GDS, Geriatric Depression Scale; SES, socioeconomic status.

Table 3 shows the results of the Poisson regression analyses. In Model 1 (which controlled for sex, age, marital status, and social support), there were significant associations between pension types and happiness. Compared to subjects with high CP Pension benefits, the PRs (95% CI) of happiness for subjects with no pension, low NA Pension benefits, and moderate EM Pension benefits were 0.67 (0.64–0.71), 0.88 (0.87–0.89), and 0.92 (0.91–0.93), respectively. In Model 2 (which also included the SES indicators), the PRs (95% CI) of happiness for subjects with no pension, low NA Pension benefits, and moderate EM Pension benefits were 0.77 (0.73–0.81), 0.95 (0.94–0.97), and 0.98 (0.97–0.99), respectively. Although the associations between pension types and happiness had attenuated slightly in Model 2, they remained significant even after adjusting for the SES indicators. Finally, in Model 3 (which further adjusted for depressive symptoms), the PRs (95% CI) of happiness for subjects with no pension, low NA Pension benefits, and moderate EM Pension benefits were 0.85 (0.81–0.89), 0.99 (0.97–1.00,) and 0.99 (0.98–1.00), respectively. The low and moderate pension types were no longer significantly associated with happiness.

**Table 3:** Adjusted prevalence ratios with 95% confidence intervals for happiness in Japanese older adults derived from Poisson regression analyses (n = 120152).

	Model 1			Model 2			Model 3		
	PR	95%CI	p-value	PR	95%CI	p-value	PR	95%CI	p-value
Sex									
Male	0.91	(0.90,0.92)	<0.001	0.90	(0.89,0.91)	<0.001	0.92	(0.91,0.93)	<0.001
Female	1.00			1.00			1.00		
Age									
65–69	1.00			1.00			1.00		
70–79	0.98	(0.97,0.99)	<0.001	1.02	(1.01,1.02)	0.001	1.01	(1.00,1.02)	0.052
80–89	1.07	(1.05,1.08)	<0.001	1.11	(1.09,1.12)	<0.001	1.12	(1.11,1.13)	<0.001
≥90	1.13	(1.10,1.17)	<0.001	1.18	(1.14,1.22)	<0.001	1.23	(1.20,1.27)	<0.001
Marital Status									
Married	1.00			1.00			1.00		
Widowed	0.94	(0.93,0.95)	<0.001	0.96	(0.95,0.97)	<0.001	0.97	(0.96,0.98)	<0.001
Divorced	0.76	(0.74,0.79)	<0.001	0.83	(0.81,0.86)	<0.001	0.86	(0.84,0.89)	<0.001
Never married	0.80	(0.77,0.83)	<0.001	0.84	(0.81,0.87)	<0.001	0.85	(0.82,0.88)	<0.001
Others	0.74	(0.69,0.79)	<0.001	0.81	(0.76,0.87)	<0.001	0.86	(0.81,0.91)	<0.001
Missing	0.88	(0.85,0.91)	<0.001	0.95	(0.92,0.98)	0.003	0.97	(0.94,1.00)	0.071
Equivalent income									
<2.00 million				1.00			1.00		
2.00–3.99 million yen				1.12	(1.11,1.13)	<0.001	1.08	(1.07,1.09)	<0.001
4.00 million yen or above				1.19	(1.18,1.21)	<0.001	1.13	(1.12,1.15)	<0.001
Missing				1.09	(1.08,1.11)	<0.001	1.07	(1.06,1.08)	<0.001
Wealth									
<0.5 million				1.00			1.00		
0.50–0.99 million yen				1.09	(1.05,1.14)	<0.001	1.04	(1.00,1.08)	0.051
1.00–4.99 million yen				1.21	(1.17,1.25)	<0.001	1.12	(1.09,1.15)	<0.001
5.00–9.99 million yen				1.27	(1.23,1.31)	<0.001	1.16	(1.12,1.19)	<0.001
10.00–49.99 million yen				1.42	(1.37,1.46)	<0.001	1.26	(1.22,1.29)	<0.001
≧50.00 million yen				1.52	(1.47,1.56)	<0.001	1.32	(1.29,1.36)	<0.001
Missing				1.26	(1.22,1.30)	<0.001	1.16	(1.12,1.19)	<0.001
Education									
≦9 years				1.00			1.00		
10–12 years				1.05	(1.04,1.06)	<0.001	1.03	(1.03,1.04)	<0.001
≧13 years				1.10	(1.09,1.11)	<0.001	1.08	(1.07,1.09)	<0.001
Missing				1.02	(0.98,1.05)	0.350	1.02	(0.99,1.05)	0.254
Working Status									
Never worked				1.00			1.00		
Working now				1.04	(1.02,1.05)	<0.001	0.99	(0.97,1.01)	0.251
Retired				0.99	(0.98,1.01)	0.518	0.99	(0.98,1.01)	0.281
Missing				0.99	(0.97,1.01)	0.360	0.98	(0.96,1.00)	0.075
Occupation									
Profession, Engineer, Manager				1.00			1.00		
Clerical worker/sales/service jobs				0.98	(0.97,0.99)	<0.001	0.99	(0.98,1.00)	0.056
Labor				0.93	(0.92,0.95)	<0.001	0.95	(0.94,0.97)	<0.001
Agriculture, forestry or fisheries/self-employed				0.97	(0.95,0.98)	<0.001	0.98	(0.97,1.00)	0.026
Others				0.93	(0.92,0.95)	<0.001	0.95	(0.94,0.97)	<0.001
Never worked				0.99	(0.97,1.01)	0.431	1.00	(0.98,1.03)	0.719
Missing				0.97	(0.95,0.99)	<0.001	0.98	(0.97,1.00)	0.031
Pension Types									
CP Pension(Rich)	1.00			1.00			1.00		
EM Pension(Moderate)	0.92	(0.91,0.93)	<0.001	0.98	(0.97,0.99)	<0.001	0.99	(0.98,1.00)	0.267
NA Pension(Poor)	0.88	(0.87,0.89)	<0.001	0.95	(0.94,0.97)	<0.001	0.99	(0.97,1.00)	0.050
No Pension(Zero)	0.67	(0.64,0.71)	<0.001	0.77	(0.73,0.81)	<0.001	0.85	(0.81,0.89)	<0.001
GDS									
No depression(GDS<5)							1.00		
Moderate depression(5≦GDS<10)							0.56	(0.55,0.56)	<0.001
Depression(≧10)							0.18	(0.17,0.19)	<0.001
Missing							0.78	(0.77,0.79)	<0.001
Receiving emotional support									
Present	1.00			1.00			1.00		
Absent	0.79	(0.77,0.82)	<0.001	0.80	(0.78,0.82)	<0.001	0.85	(0.83,0.87)	<0.001
Missing	1.03	(1.00,1.07)	0.066	1.03	(0.99,1.07)	0.094	1.02	(0.99,1.06)	0.237
Providing emotional support									
Present	1.00			1.00			1.00		
Absent	0.83	(0.81,0.85)	<0.001	0.85	(0.83,0.87)	<0.001	0.93	(0.91,0.95)	<0.001
Missing	0.92	(0.90,0.95)	<0.001	0.95	(0.92,0.98)	0.001	0.98	(0.95,1.01)	0.171
Receiving instrumental support									
Present	1.00			1.00			1.00		
Absent	0.63	(0.60,0.65)	<0.001	0.65	(0.62,0.67)	<0.001	0.75	(0.73,0.78)	<0.001
Missing	0.89	(0.85,0.92)	<0.001	0.90	(0.86,0.93)	<0.001	0.91	(0.88,0.95)	<0.001
Providing instrumental support									
Present	1.00			1.00			1.00		
Absent	0.98	(0.97,0.99)	0.003	0.99	(0.98,1.00)	0.036	1.00	(0.99,1.01)	0.495
Missing	0.97	(0.95,0.99)	0.001	1.00	(0.98,1.02)	0.695	1.01	(0.99,1.03)	0.178
Constant	0.83	(0.82,0.84)	<0.001	0.52	(0.50,0.54)	<0.001	0.67	(0.64,0.69)	<0.001

Abbreviations: CI, confidence intervals; GDS, Geriatric Depression Scale; PR, prevalence ratio.

In the ordinary least-squares method (ranging from 1 to 10 points), pension types were associated with happiness; this association, however, was weakened by adjusting for GDS.; this association, however, is weakened by adjusting for GDS. Furthermore, the results were robust even if the effects of pension types were allowed by an intersection term with a dummy variable of retirement.

## Discussion

To the best of our knowledge, this is the first study to examine the relationship between pension types and happiness using data from a large-scale study. Using JAGES project data from 2013, we found that the higher pension types were associated with higher levels of happiness. The associations remained significant even after adjusting for SES, but lost their significance after including depressive symptoms as a covariate.

The associations between pension types and happiness were in accordance with expectations, as pension types with higher benefits may be indicative of higher SES during both working age and after retirement. Higher SES can reduce poverty, economic anxiety, and depression, thereby leading to increases in happiness.

Many previous studies have focused on income, education level, and occupation as SES indicators[1–4]. However, pension types may be more indicative of long-term SES as they can reflect a person’s job history over their life course. We controlled for equivalent income and wealth as indicators of SES in older adults in Model 2, and also controlled for GDS to indicate working-age SES in Model 3. We had postulated that the associations between pension types and happiness would be completely diminished after adjusting for SES due to the close relationship between pension types and SES. Contrary to our expectations, the associations remained significant (albeit slightly weakened) even after adjusting for SES. Although pension types also reflect SES, they are not simple proxies of conventional SES indicators. In this study, the pension types were found to be stand-alone indicators of happiness. Pension types may be associated with happiness independently of conventional SES indicators because pension benefits can function as insurance for reducing old-age economic risks and associated anxiety.

Many older people have limited income and experience poverty caused by disease or unemployment. Due to the uncertainties of life expectancy, the use of personal savings for living expenses at an older age is accompanied by the risk of prematurely exhausting all available funds. As a result, these individuals are susceptible to stress and anxiety after retirement. On the other hand, pensions provide a fixed income to older pensioners until death. In Japan, pension benefits account for approximately 70% of all income for older people, and there are wide gaps in pension benefits among the different pension types. Therefore, the amount of pension benefits can be a major contributing factor to economic anxiety in older adults.

It has been reported that low income was associated with depressive symptoms [38], and that depressive symptoms were associated with low happiness [17,18,39–42]. Furthermore, pension types reflect about 70% of income levels in older Japanese. Pension coverage have been shown to reduce geriatric depression [43]. Those findings suggest that the association between higher pension benefits and happiness may be due to the reduction of poverty, economic anxiety, and especially depressive symptoms. In our analysis, this association weakened substantially after further adjusting for GDS, which may have been influenced by the relationship between pension types and depressive symptoms.

### Limitations

This study has several limitations that should be acknowledged. First, this analysis adopted a cross-sectional design, and causal relationships between pension types and happiness could not be determined. However, happiness is unlikely to affect pension types, whereas pension types may influence happiness. We plan to employ a cohort study approach (using the 2013-2016/2017 JAGES data) to explore such potential causality. Second, the happiness measure was based on a single item and was self-reported. Nevertheless, this measure has been commonly used in previous studies and has shown to be moderately reliable [25–27]. More objective and multiple-item measures of happiness should be considered for further studies. Third, pension benefits were also considered to be associated with use of services such as health services, social support, housing, and the bus or train. Future analyses should include these factors.

Finally, our survey sample included older Japanese participants who had not been certified as needing long-term care services, so assuming that the happiness of those requiring long-term care services is lower (due to poorer health), happiness may be overrated in our analysis, and our findings may therefore have limited generalizability to Japanese older people with more severe conditions.

## Conclusions

In summary, we performed Poisson regression analyses using JAGES data to calculate PRs of happiness after controlling for various covariates. The association between pension types and happiness weakened slightly after controlling for SES, but remained significant. In addition to SES, pension types should also be considered when assessing the determinants of happiness in older adults.
